# Prevalence and Antimicrobial Resistance of *Escherichia coli* Isolated from Various Meat Types in the Tamale Metropolis of Ghana

**DOI:** 10.1155/2020/8877196

**Published:** 2020-11-12

**Authors:** Frederick Adzitey, Prince Assoah-Peprah, Gabriel A. Teye, Anou M. Somboro, Hezekiel M. Kumalo, Daniel G. Amoako

**Affiliations:** ^1^Department of Veterinary Science, University for Development Studies, P.O. Box TL 1882, Tamale, Ghana; ^2^Department of Animal Science, University for Development Studies, P.O. Box TL 1882, Tamale, Ghana; ^3^Antimicrobial Research Unit, University of KwaZulu-Natal, Durban 4000, South Africa; ^4^Discipline of Medical Biochemistry, University of KwaZulu-Natal, Durban, South Africa

## Abstract

Meats are important potential sources of foodborne pathogens including *Escherichia coli*. This study was conducted to determine the prevalence and antimicrobial resistance of *Escherichia coli* isolated from meats in the Tamale metropolis of Ghana. Isolation of *Escherichia coli* was done using the procedure according to the USA-FDA *Bacteriological Analytical Manual*. Antibiotic resistance patterns in the *Escherichia coli* isolates were determined by the Kirby-Bauer disk diffusion method against 8 antibiotics. The overall prevalence of *Escherichia coli* in the meat samples was 84.00% (189/225). Mutton (88.89%), guinea fowl (88.89%), beef (86.67%), local chicken (80.00%), and chevon (75.56%) were contaminated by *Escherichia coli*. The average coliform count was 4.22 cfu/cm^2^ and was highest in guinea fowl (4.94 log cfu/cm^2^) and lowest in local chicken (3.23 log cfu/cm^2^). The *Escherichia coli* isolates were highly resistant to erythromycin (85.00%), tetracycline (73.33%), and ampicillin (71.67%). The multiple antibiotic resistance (MAR) index ranged from 0.13 to 1. The *Escherichia coli* isolates exhibited 23 antimicrobial resistance patterns with resistant pattern TeAmpE (tetracycline-ampicillin-erythromycin) being the most common. Multidrug resistance was 68.33% (41/60) among the *Escherichia coli* isolates. The results showed that *Escherichia coli* was commonly present in the various meat types and exhibited multidrug resistances, necessitating efficient antibiotic stewardship guidelines to streamline their use in the production industry.

## 1. Introduction

Meat is consumed by many people as an important source of protein and other nutrients [1]. It has been estimated that 62 billion chickens, 1.5 billion pigs, 545 million sheep, 444 million goats, and 301 million cattle were slaughtered for meat consumption worldwide in 2014 [2]. Furthermore, pork is the most consumed meat (average consumption of 16 kg per year in 2013), followed by poultry (15 kg), beef/buffalo (9 kg), and mutton and chevon (2 kg) [2]. Meat consumption is known to be highest across high-income countries and lowest in low-income countries [2, 3]. Speedy [3] reported that the United States of America is the leading consumer of meat in the world with 124 kg/capita/year. Africa and South Asia are the least consumers of meat with a consumption of between 3 and 5 k/capita/year [3].

Most meats have high water content corresponding to a water activity of approximately 0.99 which is suitable for microbial growth [4]. Microbial growth can lead to food spoilage and foodborne infections in humans, resulting in economic and health losses [5]. Some strains of *Escherichia coli* (*E. coli*) are among the pathogens that have been associated with foodborne infections in humans. Some of the foodborne infections in humans have also occurred as a result of the consumption of contaminated meats. For instance, the Centers for Disease Control and Prevention [6] reported an outbreak of *E. coli* infections linked to the consumption of ground beef which resulted in 29 hospitalizations and 0 death. A more serious *E. coli* outbreak linked to the consumption of ground beef occurred in 2018 which led to 1 death and 6 hospitalizations [7]. In the European Union, 6,073 confirmed cases of Shiga toxin-producing *E. coli* (STEC) infections were reported in 2017 [8]. There were 20 deaths (case fatality of 0.5%), and the contribution of STEC from animal sources was found [8].

Although most foodborne infections are self-limited, antimicrobials are used when necessary. The use of antimicrobials has resulted in the development of resistant pathogens including *E. coli*, which is a threat to public health. To accurately study the involvement of microorganisms in foodborne infections, robust tools/methods that will ensure effective isolation, phenotypic, and/or genetic characterization are required. Research has demonstrated that meat samples in Ghana are contaminated by *E. coli* [9–15]. However, a study comparing *E. coli* in different meat types and their resistance pattern is limited in the Tamale metropolis. Therefore, this study was carried out to determine the prevalence and antimicrobial resistance of *E. coli* isolated from various meat types in the Tamale metropolis of Ghana.

## 2. Materials and Methods

### 2.1. Location of Study

This study was carried out at the Tamale metropolis of Ghana. The metropolis lies in between latitude 9° 16 and 9° 34 north and longitudes 0° 36 and 0° 57 west, with a total estimated land size of 646.90180 sqkm [16]. It shares boundaries with Sanarigu District to the west and north, Mion District to the east, East Gonja to the south, and Central Gonja to the southwest.

### 2.2. Sample Collection

A total of two hundred and twenty-five (225) meat samples comprising of beef (*n* = 45), chevon (*n* = 45), mutton (*n* = 45), chicken (*n* = 45), and guinea fowl (*n* = 45) were sampled. Sterile cotton swabs were used to swab an area of 10 cm^2^ of each meat samples. The surfaces of carcasses displayed for sale were randomly swabbed. A sterile sampling template of 10 cm^2^ was placed on the surface of the meat, and a sterile swab was used to swab the entire surface of the area demarcated by the sampling template. The swabs were transported at 4°C and analyzed immediately upon reaching the laboratory for *Escherichia coli* and coliforms.

### 2.3. Isolation of *Escherichia coli*

The procedure according to the Food and Drug Administration *Bacteriological Analytical Manual* [17] slightly modified as reported by Adzitey [9] was used. In brief, the swabs were dipped into 10 ml Buffered Peptone Water and incubated at 37°C for 24 h. After which, 0.1 ml of the aliquots were streaked on Levine's Eosin-Methylene Blue Agar and incubated at 37°C for 24 h. Presumptive *E. coli* colonies appeared as dark centered and flat, with or without a metallic sheen. Presumptive *E. coli* colonies were purified on Trypticase Soy Agar and incubated at 37°C for 24 h. They were identified and confirmed using Gram staining, growth on MacConkey Agar, growth in Brilliant Green Bile Broth, and *E. coli* latex agglutination test. All media and reagents used were purchased from Oxoid Limited, Basingstoke, UK.

### 2.4. Analysis of Meat Samples for Coliforms

Coliform was determined using a modified method of Maturin and Peeler [18] and Adzitey et al. [19]. Swab samples were dipped into 25 ml universal bottles containing 10 ml of 1% Buffered Peptone Water. 10-fold serial dilutions from 10^−1^ to 10^−5^ were performed using 1 ml from each dilution. Approximately 100 *μ*l of the aliquots was spread plated onto MacConkey Agar (Oxoid Limited, Basingstoke, UK). The MacConkey Agar plates were incubated at 37°C for 24 h and counted with a colony counter. Coliform count was calculated using the formula [18]

(1) $$\begin{array}{c} N=\frac{\sum C}{\left(1*n_{1}\right)+\left(0.1*n_{2}\right)*\left(d\right)}, \end{array}$$

where *N* is the number of colonies per cm^2^, ∑*C* is the sum of all colonies on all plates counted, *n*_1_ is the number of plates in the first dilution counted, *n*_2_ is the number of plates in the second dilution counted, and *d* is the dilution from which the first counts were obtained.

### 2.5. Antimicrobial Susceptibility Test and Determination of Multiple Antibiotic Resistance

An antimicrobial susceptibility test was done according to the disk diffusion method [20]. A total of 60 *E. coli* isolates were subjected to an antimicrobial susceptibility test using the following antibiotics: ampicillin (10 *μ*g), ceftriaxone (30 *μ*g), chloramphenicol (30 *μ*g), ciprofloxacin (5 *μ*g), erythromycin (15 *μ*g), gentamicin (10 *μ*g), sulphamethoxazole/trimethoprim (22 *μ*g), and tetracycline (30 *μ*g). Pure colonies of *E. coli* were inoculated in Trypticase Soy Broth (Oxoid Limited, Basingstoke, UK) and incubated at 37°C for 18 h. The turbidity was adjusted to 0.5 McFarland standard using sterile Trypticase Soy Broth and spread plated on Müller Hinton Agar (Oxoid, Basingstoke, UK). Four antibiotic disks were placed on the surface of the Müller Hinton Agar at a distance to avoid overlapping of inhibition zones. They were then incubated at 37°C for 24 h. After incubation, the inhibition zones were measured, and the results were interpreted using the CLSI protocol [21]. The number of antibiotics each bacterium was resistant to in the disk diffusion test was noted for identification of multidrug-resistant (MDR) strains. Isolates showing resistance to ≥1 agent in >3 antibiotic classes were considered MDR [22]. The multiple antibiotic resistance (MAR) index was calculated and interpreted according to Krumperman [23] using the formula *a*/*b*, where “*a*” represents the number of antibiotics to which a particular isolate was resistant and “*b*” is the total number of antibiotics tested.

### 2.6. Statistical Analysis

The prevalence data for *E. coli* was analyzed using a binary logistic generalized linear model of Statistical Package for Service Solutions Program Version 20.0. Statistical difference was done using Wald chi square, and means were separated at the 5% significant level. Coliform counts were analyzed using GenStat Release 12 Edition, and one-way analysis of variance was used to test the significant difference at *p* < 0.05.

## 3. Results and Discussion

### 3.1. Prevalence of *Escherichia coli* and Total Coliform Counts in the Various Meat Types

The occurrence of *E. coli* and total coliform counts in the various meat types are presented in Table 1. *E. coli* were found in guinea fowl 40 (88.89%), mutton 40 (88.89%), beef 39 (86.67%), local chicken 36 (80.00%), and chevon 34 (75.56%). There were no significant differences (*p* > 0.05) among the various meat types. Nonetheless, guinea fowl and mutton were most contaminated, followed by beef, local chicken, and chevon. The contamination of the meat samples by *E. coli* indicates that lapses occurred during the slaughtering of the animals and transportation and selling of the meats [2]. This is because the muscle of a nondiseased life animal is indispensably sterile. Once the animal is slaughtered, the muscles are exposed and can be contaminated by microorganisms. *E. coli* are known to naturally harbour in the gastrointestinal tract of farm animals [17]. They cross-contaminate meats when the gastrointestinal tract ruptures during evisceration. It was observed during sampling that knives used for cutting meats were not sterilized intermittently. The tables also had remains of meat exudates and particles from previous use. All these posed as potential sources for cross-contamination of the meats by *E. coli*. A similar observation was made by [24] among meat sellers in the Accra metropolis. The knives and tables could harbour *E. coli* which cross-contaminated the meats. Therefore, some measures as described by Adzitey [25] must be adapted to control and prevent bacterial foodborne infections from the consumption of the various meat types.

Rasmussen et al. [13] examined locally produced chicken meat and imported chicken thighs into Ghana for *E. coli* and observed that the local chickens 36 (64.29%) and imported chickens 73 (55.30%) were contaminated by *E. coli*. Adzitey [9] also detected 56% (39/70) of *E. coli* in beef samples sold in the Tamale metropolis of Ghana. *E. coli* were observed in beef, pork, and fresh and grilled guinea fowls in the Bolgatanga municipality of Ghana [11, 12]. *E. coli* were not found in beef and chicken samples collected from three administrative regions (Gyeonggi, Gyeongsang, and Chungchong) of Korea [26]. Of 119 chicken meats sampled in the city of Taif, Saudi Arabia, 31.1% showed contamination with *E. coli* [27]. In Bhaktapur Metropolitan City of Nepal, *E. coli* were detected in 33 (33.00%) of chicken meats [28]. In the United States of America, Zhao et al. [29] reported that 83.5% of chicken breasts were contaminated with *E. coli*. The findings of Zhao et al. [29] were similar to this study; however, lower contamination rates were reported by [9, 13, 27, 28].

The total coliform counts were 4.94 log cfu/cm^2^ for guinea fowl, 4.72 log cfu/cm^2^ for chevon, 4.39 log cfu/cm^2^ for mutton, 3.81 log cfu/cm^2^ for beef, and 3.23 log cfu/cm^2^ for local chicken. Thus, it was highest for guinea fowl, followed by chevon, mutton, beef, and local chicken. Howbeit, statistical differences (*p* > 0.05) were not observed among the meat types. Coliforms include *Citrobacter*, *Enterobacter*, *Hafnia*, *Klebsiella*, and *Escherichia coli* species, and the detection of coliforms in the meat samples is an indication of faecal contamination or processing under an unsanitary environment [17]. Kim and Yim [26] reported an average coliform count of 0.37 log cfu/g in meat samples collected from Gyeonggi, Gyeongsang, and Chungchong in Korea. The coliform counts were 0.30 ± 0.78 and 1.03 ± 1.28 for beef and chicken, respectively [26]; this study found higher coliform counts in the meat samples examined. In Kumasi, Ghana, Antwi-Agyei and Maalekuu [30] recorded total coliform counts of 3.52 × 10^7^ cfu/g (7.55 log cfu/g) for goat meat (chevon) and 2.14 × 10^7^ cfu/g (7.33 log cfu/g) for cattle meat (beef), which were higher than the present study. Maharjan et al. [31] reported that more than 80% of meat samples collected from Kathmandu, Nepal, had coliform bacteria.

### 3.2. Phenotypic Antimicrobial Susceptibility Testing of Escherichia coli

The phenotypic antimicrobial resistance of the 60 *E. coli* isolates is shown in Tables 2 and 3. The *E. coli* isolates were highly resistant to erythromycin (85.00%), tetracycline (73.33%), and ampicillin (71.67%) but susceptible to gentamicin (88.33%), ciprofloxacin (85.00%), sulphamethoxazole/trimethoprim (85.00%), chloramphenicol (83.33%), and ceftriaxone (80.00%). Intermediate resistance was observed for all the antibiotics examined, and it ranged from 3 to 10%. The *E. coli* of meat origin being resistant to antimicrobials can be linked to their use in animal production. Residues from these antimicrobials can also be deposited in meats which can be transferred into humans when consumed. The overall consequence is humans not responding to antimicrobial treatments due to the presence of resistant strains or residues in them. In Ghana, antibiotics are mainly used as prophylactics and treatment of sick animals, rather than as growth promoters. Ekli et al. [1] reported that antimicrobials including ciprofloxacin (32.0%), sulphamethoxazole/trimethoprim (17.1%), gentamicin (1.8%), ceftriaxone (0.9%), chloramphenicol (0.9%), and tetracycline (0.9%) were used by farmers in Wa, municipality of Ghana, as prophylactics or to treat animal diseases. They also indicated that the farmers (73.2%) did not observe withdrawal periods when they administer, or antimicrobials are administered to their animals before sales or slaughter. These prone bacteria of these animals to develop resistance to antimicrobials and deposition of antimicrobial residues in their muscle tissues.

Adzitey [10] observed that *E. coli* isolated from beef in Techiman municipality, Ghana, were resistant to tetracycline (44.44%), erythromycin (68.89%), and chloramphenicol (44.44%), but susceptible to ciprofloxacin (95.56%), sulphamethoxazole/trimethoprim (82.22%), and gentamicin (75.56%). Resistance to tetracycline and erythromycin but not chloramphenicol was higher in the present study compared with Adzitey [10].

Similarly, high susceptibility to ciprofloxacin, sulphamethoxazole/trimethoprim, and gentamicin was found in both studies. Also, in Ghana, Rasmussen et al. [13] reported that *E. coli* from locally produced chicken meats were resistant to tetracycline (88.9%) and ampicillin (69.4%), while those from imported chicken meats were resistant to tetracycline (57.5%) and ampicillin (61.6%). Resistance to ampicillin in locally produced chicken meat was similar to the current study but not the rest. Saud et al. [28] found that *E. coli* isolated from chicken meats in Bhaktapur Metropolitan City, Nepal, were resistant to gentamicin (24.2%) and tetracycline (60.6%), which contradicts this study. *E. coli* from chicken meats in Indonesia were resistant to tetracycline (79.24%) and chloramphenicol (9.43%) [32], which were similar to this study. Altalhi et al. [27] observed that *E. coli* isolated from retail raw chicken meat in Taif, Saudi Arabia, were resistant to ampicillin (78.4%), chloramphenicol (32.4%), and gentamicin (24.3%). Resistance to ampicillin was similar to this study but lower for chloramphenicol and gentamicin. Martínez-Vázquez et al. [33] reported that *E. coli* from retail meats in Tamaulipas, Mexico, were resistant to ampicillin (92%) and tetracycline (75%), which were comparable to this study.

The multiple antibiotic (MAR) index ranged from 0.13 (resistant to one antibiotic) to 1.0 (resistant to eight antibiotics) (Table 3). Bacteria having MARindex > 0.2 originate from a high-risk source of contamination where several antibiotics or growth promoters are used while values < 0.2 show bacteria from the source with less antibiotic use [34, 35]. A completely resistant isolate has an MAR index of 1.0. The *E. coli* isolates were resistant to one (13.33%), two (16.67%), three (41.67%), four (13.33%), and five (8.33%) antimicrobials. Resistance to zero, six, seven, and eight antimicrobials was 1.67% each. The *E. coli* isolates also exhibited twenty-three (23) different resistance patterns. The resistance pattern TeAmpE (tetracycline-ampicillin-erythromycin) was the most common and was exhibited by sixteen isolates. Most of the *E. coli* isolates exhibited an MAR index of ≥0.25 reflecting a greater resistance to the group of antimicrobial agents studied. This means that there is greater antimicrobial use in production on the farms the animals were reared, which needs the attention of all relevant stakeholders in Ghana. Furthermore, *E. coli* isolates of meat origin with an MAR index of 0.4 and above are associated with human faecal contamination, while an MAR index of less than 0.4 is associated with nonhuman faecal contamination [36]. Based on this assumption, 26.7% of the samples were human faecal contamination and the rest were not. It has been reported that meat sellers/butchers at Tamale markets do not adhere to strict hygiene in the sale of meat, and this could possibly contribute to faecal contamination (Adzitey et al. [37]). Similarly to this work, Adzitey [10] showed that *E. coli* isolated from beef in Techiman exhibited twenty-five (25) resistance patterns, and the MAR index ranged from 0.11 to 0.78. Adzitey [10] also found that majority of *E. coli* isolates were resistant to three antimicrobials (14 isolates), followed by four antimicrobials (13 isolates). In addition, three and one isolates were resistant to 5 and 7 antimicrobials, respectively.

Multidrug resistance (MDR), that is, resistant to 3 or more different classes of antimicrobials, was observed in 41 (68.33%) of the isolates. Multidrug-resistant *E. coli* can be transferred from one carcass to the other and finally consumed by humans. Multidrug resistance is a cause for concern due to the fact that it limits therapeutic options available for animal and human treatment. *E. coli* isolates of meat origin exhibiting multidrug resistance with susceptible ones serve as sources of resistant genes and increase the chances for the transfer of resistance genes to those that are sensitive. In Cape Coast, Ghana, Anning et al. [15] reported that 4.8% of *E. coli* from meat sources were multidrug-resistant to cefuroxime-chloramphenicol-ampicillin. Altalhi et al. [27] found that *E. coli* of raw chicken meat were resistant to one or more antimicrobials. They also found that 86.5% were resistant to at least one antimicrobial and 40.5% of the isolates were resistant to at least three antimicrobials. Saud et al. [28] observed 52.5% multidrug resistance in *E. coli* isolates of meat origin (chicken and buffalo meat). In addition, they found overall multidrug resistance of 69.81%, and resistance to zero, one, two, three, four, five, and six antibiotics was 13.21%, 16.98%, 33.96%, 15.09%, 20.75%, 0.00%, and 0.00%, respectively [30]. In Tamaulipas, Mexico, Martínez-Vázquez et al. [33] detected that 92.4% of *E. coli* obtained from retail meats exhibited multiresistance.

## 4. Conclusions

Overall, 189 (84.00%) of the meat samples were positive for *Escherichia coli*, and the overall total coliform counts were 4.22 log cfu/cm^2^. Contamination of the meat samples by *Escherichia coli* and coliforms did not differ significantly (*p* < 0.05) from each other. Phenotypic characterization revealed a high resistance to ampicillin, erythromycin, and tetracycline but susceptibility to ceftriaxone, chloramphenicol, ciprofloxacin, gentamicin, and sulfamethoxazole/trimethoprim. The high resistance of the *Escherichia coli* isolates of meat origin to the various antibiotics observed requires that farmers should use less antibiotics in animal production. They should rely on good management practices to prevent the occurrence of diseases that will necessitate the use of antibiotics. Further research will involve the use of molecular characterization to determine resistant genes, virulence, and whole-genome sequencing.

## Figures and Tables

**Table 1:** Prevalence of *Escherichia coli* and coliform counts in meat samples sold at the Tamale Metropolis.

Sample	No. of samples examined	^a^No. (%) positive	Coliforms (log cfu/cm^2^)
Beef	45	39 (86.67)	3.81 ^∗^(3.48-4.14)
Chevon	45	34 (75.56)	4.72 (4.09-5.35)
Mutton	45	40 (88.89)	4.39 (4.25-4.53)
Local chicken	45	36 (80.00)	3.23 (3.16-3.30)
Guinea fowl	45	40 (88.89)	4.94 (4.65-5.24)
Overall	225	189 (84.00)	4.22 (3.16-5.35)

^a^No.: number of samples positive for *Escherichia coli*; ^∗^range values for coliform counts.

**Table 2:** Percentage antibiotic resistance of *Escherichia coli* isolated from meat samples in Tamale metropolis.

Antimicrobial	*R*	*I*	*S*	*R* (%)	*I* (%)	*S* (%)
Ampicillin (Amp) 10 *μ*g	≤13	14-16	≥17	71.67	10.00	18.33
Ciprofloxacin (Cip) 5 *μ*g	≤15	16-20	≥21	8.33	6.67	85.00
Ceftriaxone (Cro) 30 *μ*g	≤19	20-22	≥23	16.67	3.33	80.00
Chloramphenicol (C) 30 *μ*g	≤12	13-17	≥18	10.00	6.67	83.33
Erythromycin (E) 15 *μ*g	≤13	14-22	≥23	85.00	10.00	5.00
Gentamicin (Cn) 10 *μ*g	≤12	13-14	≥15	6.67	5.00	88.33
Sulphamethoxazole/trimethoprim (Sxt) 25 *μ*g	≤10	11-15	≥16	8.33	6.67	85.00
Tetracycline (Te) 30 *μ*g	≤11	12-14	≥15	73.33	6.67	25.00
Overall (%)				37.71	6.04	56.25

*S*: susceptible; *I*: intermediate; *R*: resistant.

**Table 3:** Antibiotic resistance profile and multiple antibiotic resistance index of individual *Escherichia coli* isolated from meat samples in Tamale Metropolis.

Serial number	*Escherichia coli* code	Source	Antibiotic-resistant profile	Number of antibiotics	MAR index
1	CC15	Chevon		0	0.00
2	AM13	Mutton	Amp	1	0.13
3	NB1	Beef	E	1	0.13
4	CB1	Beef	E	1	0.13
5	CC2	Chevon	E	1	0.13
6	NB15	Beef	E	1	0.13
7	NC10	Chevon	E	1	0.13
8	NLC5	Local chicken	E	1	0.13
9	Cg3	Guinea fowl	Te	1	0.13
10	NC3	Chevon	AmpE	2	0.25
11	CM11	Mutton	AmpE	2	0.25
12	CM15	Mutton	AmpE	2	0.25
13	NM3	Mutton	AmpE	2	0.25
14	AC10	Chevon	TeAmp	2	0.25
15	CM4	Mutton	TeCro	2	0.25
16	Cg5	Guinea fowl	TeE	2	0.25
17	Cg15	Guinea fowl	TeE	2	0.25
18	NLC15	Local chicken	TeE	2	0.25
19	Tg14	Guinea fowl	TeE	2	0.25
20	AB7	Beef	AmpCCn	3	0.38
21	AM1	Mutton	AmpECn	3	0.38
22	NB8	Beef	AmpECro	3	0.38
23	CM1	Mutton	TeAmpCn	3	0.38
24	NC1	Chevon	TeAmpCro	3	0.38
25	AC15	Chevon	TeAmpE	3	0.38
26	AM14	Mutton	TeAmpE	3	0.38
27	CB4	Beef	TeAmpE	3	0.38
28	CB9	Beef	TeAmpE	3	0.38
29	CB13	Beef	TeAmpE	3	0.38
30	CC6	Chevon	TeAmpE	3	0.38
31	CC10	Chevon	TeAmpE	3	0.38
32	NB12	Beef	TeAmpE	3	0.38
33	NM7	Mutton	TeAmpE	3	0.38
34	Cg9	Guinea fowl	TeAmpE	3	0.38
35	Sg1	Guinea fowl	TeAmpE	3	0.38
36	Sg15	Guinea fowl	TeAmpE	3	0.38
37	Tg9	Guinea fowl	TeAmpE	3	0.38
38	TLC1	Local chicken	TeAmpE	3	0.38
39	TLC4	Local chicken	TeAmpE	3	0.38
40	TLC10	Local chicken	TeAmpE	3	0.38
41	NLC3	Local chicken	TeSxtE	3	0.38
42	SLC11	Local chicken	TeSxtE	3	0.38
43	SLC15	Local chicken	TeSxtE	3	0.38
44	TLC13	Local chicken	TeSxtE	3	0.38
45	AB1	Beef	AmpCipCroC	4	0.50
46	AM9	Mutton	TeAmpECro	4	0.50
47	AB13	Beef	TeAmpSxtE	4	0.50
48	NC15	Chevon	TeAmpSxtE	4	0.50
49	Sg6	Guinea fowl	TeAmpSxtE	4	0.50
50	Sg9	Guinea fowl	TeAmpSxtE	4	0.50
51	SLC2	Local chicken	TeAmpSxtE	4	0.50
52	SLC6	Local chicken	TeAmpSxtE	4	0.50
53	NM8	Mutton	TeAmpCipSxtE	5	0.63
54	Tg5	Guinea fowl	TeAmpSxtEC	5	0.63
55	AC7	Chevon	TeAmpSxtECro	5	0.63
56	AB15	Beef	TeAmpSxtECro	5	0.63
57	Tg1	Guinea fowl	TeAmpSxtECro	5	0.63
58	NLC9	Local chicken	TeAmpCipSxtEC	6	0.75
59	AC1	Chevon	TeAmpCipSxtECroC	7	0.88
60	NM11	Mutton	TeAmpCipSxtECroCCn	8	1.00

Amp: ampicillin; Cip: ciprofloxacin; Cro: ceftriaxone; C: chloramphenicol; E: erythromycin; Cn: gentamicin; Sxt: sulphamethoxazole/trimethoprim; Te: tetracycline.

## Data Availability

All datasets on which the conclusions of the manuscript rely are presented in the paper.
